# Patient-Specific Phantomless Estimation of Bone Mineral Density and Its Effects on Finite Element Analysis Results: A Feasibility Study

**DOI:** 10.1155/2019/4102410

**Published:** 2019-01-03

**Authors:** Young Han Lee, Jung Jin Kim, In Gwun Jang

**Affiliations:** ^1^Department of Radiology, College of Medicine, 50-1 Yonsei-ro, Seodaemun-gu, Seoul 03722, Yonsei University College of Medicine, Republic of Korea; ^2^The Cho Chun Shik Graduate School of Green Transportation, 291, Daehak-ro, Yuseong-gu, Daejeon 34141, Korea Advanced Institute of Science and Technology, Republic of Korea

## Abstract

**Objectives:**

This study proposes a regression model for the phantomless Hounsfield units (HU) to bone mineral density (BMD) conversion including patient physical factors and analyzes the accuracy of the estimated BMD values.

**Methods:**

The HU values, BMDs, circumferences of the body, and cross-sectional areas of bone were measured from 39 quantitative computed tomography images of L2 vertebrae and hips. Then, the phantomless HU-to-BMD conversion was derived using a multiple linear regression model. For the statistical analysis, the correlation between the estimated BMD values and the reference BMD values was evaluated using Pearson's correlation test. Voxelwise BMD and finite element analysis (FEA) results were analyzed in terms of root-mean-square error (RMSE) and strain energy density, respectively.

**Results:**

The HU values and circumferences were statistically significant (*p* < 0.05) for the lumbar spine, whereas only the HU values were statistically significant (*p* < 0.05) for the proximal femur. The BMD values estimated using the proposed HU-to-BMD conversion were significantly correlated with those measured using the reference phantom: Pearson's correlation coefficients of 0.998 and 0.984 for the lumbar spine and proximal femur, respectively. The RMSEs of the estimated BMD values for the lumbar spine and hip were 4.26 ± 0.60 (mg/cc) and 8.35 ± 0.57 (mg/cc), respectively. The errors of total strain energy were 1.06% and 0.91%, respectively.

**Conclusions:**

The proposed phantomless HU-to-BMD conversion demonstrates the potential of precisely estimating BMD values from CT images without the reference phantom and being utilized as a viable tool for FEA-based quantitative assessment using routine CT images.

## 1. Introduction

Osteoporosis is a common metabolic bone disorder that leads to increased bone fracture risk. As the elderly population increases, the prevalence of osteoporosis is steadily increasing [1, 2]; thus, bone strength assessment has become more important as a diagnosis tool for osteoporosis. In principle, bone strength depends on two parameters: bone quality (e.g., bone architecture) and bone quantity (e.g., bone mineral density). In clinical practice, the estimation of bone strength has been based on the representative areal bone mineral density (aBMD) for the region of interest obtained by dual energy X-ray absorptiometry (DXA) [3–5]. However, the representative aBMD cannot be a perfect stand-alone measure of bone strength because it neglects the 3D bone structure. It has been reported that two individuals who have identical bone density can have different fracture risks due to different bone structures [6].

In contrast to representative aBMDs, voxelwise volumetric BMDs (vBMDs), which can be obtained using quantitative computed tomography (QCT) [7, 8], can provide a spatial BMD distribution in 3D, thereby eliminating the aforementioned sources of errors in estimating bone strength. In order to precisely measure the BMD values, QCT examination requires a reference phantom that is constructed from K_2_HPO_4_ with known densities [9, 10]. The existence of a reference phantom eliminates potential confounding factors from scattering and beam hardening, which depend on individual patient factors such as waist circumference and weight. QCT also excludes osteophytes [11] and aortic/vascular calcifications [12], which might affect BMD. For the lumbar spine and proximal femur in which trabecular bone is prevalent, QCT can provide a diagnostic sensitivity for osteoporosis diagnosis greater than that using DXA [12].

In particular, QCT-based finite element analysis (FEA), which uses the 3D geometry and spatial BMD distribution of a target bone measured from the QCT examination, enables the precise estimation of patient-specific bone strength [13–15]. This quantitative approach has been proven to estimate fracture loads and sites [16–18]. It should be emphasized that the measurement of voxelwise BMD is critical to conduct a reliable FEA because the elastic moduli of each finite element need to be derived through the BMD-modulus relationship [12, 16–18]. For precise voxelwise BMD measurement, however, a reference phantom must be placed below the patient during scanning, thereby causing additional expenses and logistical burden in clinical imaging. Because routine CT scans (i.e., phantomless CT scans) are acquired for various purposes in clinical practice, it would be useful to be able to estimate the voxelwise BMD values using these routine CT scans without additional QCT examination.

To date, several Hounsfield unit (HU) to BMD conversion methods have been introduced [19–22]. Because BMD values are significantly related to HU values [23], the various effects of scanning protocol on HU values have been further investigated: contrast-enhanced CT [21, 22], CT colonography [24, 25], abdominal multi-detector CT [20], and spine CT [19, 22]. These studies have demonstrated that the correlation between the HU and BMD values depends on the contrast medium [25, 26], on kVp [27], and on the CT scanning regions. It is noteworthy that patient physical factors such as waist circumference must be considered for accurate phantomless HU-to-BMD conversions because these factors can affect the attenuation of radiation and therefore the HU values. However, few works in the literature to date have addressed the effects of patient physical factors on the HU-to-BMD conversion and its subsequent impact on FEA-based quantitative assessment.

Therefore, for the lumbar spine and proximal femur, this study (1) proposes the phantomless HU-to-BMD conversion equations that consider the circumference and bone area as patient-specific physical factors, (2) investigates the correlation of BMD values between the proposed phantomless conversion and the reference phantom-based measurement, and (3) analyzes the accuracy of the estimated BMD values in terms of voxelwise BMD and FEA.

## 2. Materials and Methods

### 2.1. Study Population

The study population was retrospectively identified using the hospital information system. The inclusion criteria were (1) QCT examination conducted in April 2014 and (2) no bony abnormalities on the radiologic report. For a total of 39 identified cases, the purposes of the QCT examination were medical check-up (*n*=36), breast cancer follow-up (*n*=1), and thyroid cancer follow-up (*n*=2). The gender distribution was 14 males and 25 females. The mean age was 49.1 years (age 30–73). The inclusion criteria were QCT examination and no bony abnormalities on the radiologic report. This retrospective study was approved by the hospital's Institutional Review Board (IRB).

### 2.2. Image Protocol

QCT scans were performed in a 64-channel CT (Somatom definition AS+, Siemens, Erlangen, Germany), of which quality control and assessment are routinely performed every three months. The CT scan parameters were optimized for QCT examination as follows: 120 kVp, 150 effective mAs, beam collimation = 20 mm, rotation speed = 0.6 seconds, pitch = 1.0 : 1, 512 × 512 matrix, field of view (FOV) = 360 mm, slice thickness = 3 mm, and reconstruction with B40s medium. The CARE Dose 4D and CARE kV were off. The CT dose index (CTDI 32 cm) was 10.10 mGy, and the dose length product (DLP) was 218.7 mGy·cm. Deep-inspiration breath-hold method was used for the QCT scans. All QCT scans were done with no contrast media.

### 2.3. Calculation of the Reference BMD Values Using the Reference Phantom

Five regions of interest (ROIs) for the five different mineral contents of the reference phantom (Mindways Inc., Austin, TX, USA) were recorded in the same CT images. For the CT image of each patient, the phantom-based calibration algorithm [28, 29] determined the linear correlation between the known mineral densities and their corresponding HU values as follows:

(1) $$\begin{array}{c} {\left(\text{BMD}\right)}_{\text{phantom}}=\alpha \times \left(\text{HU}\right)+\beta , \end{array}$$

where *α* and *β* are the patient-specific values that are to be determined. Thus, using equation (1) with the determined *α* and *β*, the voxelwise BMD values of the lumbar spine and hip of each patient were calculated as the ground truth.

### 2.4. Image Analysis: Evaluation of Patient Factors

All images were assessed by one musculoskeletal fellowship-trained radiologist with ten years of experience in musculoskeletal radiology. Thirty-nine QCT images of different patients were retrospectively analyzed at the middle axial levels of the L2 vertebra and hip. Quantitative assessments of the ROIs were performed using 80–100 mm^2^ drawings of the trabecular compartment of the L2 body and left hip femoral neck level, where the HU values were recorded.

**Figure 1:**
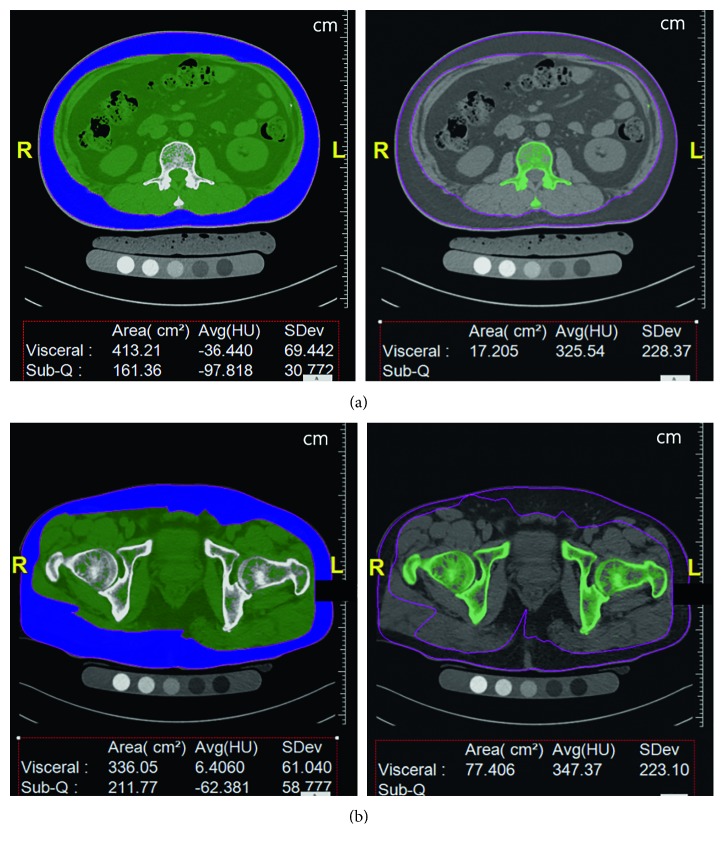
Screenshots of the semiautomatic calculation software (FatScan, N2 systems): (a) at the L2 level, the circumferences of the body and cross-sectional area of bone are segmented; and (b) at the hip level, the circumferences of the body and cross-sectional area of the bone are segmented. In this case, the circumference of the L2 level is 89.559 cm, and the cross-sectional area of L2 is 17.205 cm^2^. The circumference of the hip level is 94.858 cm, and the cross-sectional area of the hip is 77.406 cm^2^.

As can be seen in Figure 1, waist circumferences and cross-sectional areas of bones were measured at the same axial levels of the target sites; this was done using semiautomatic calculation software (FatScan, N2 systems, Osaka, Japan), which is routinely used to analyze central/peripheral fat for central obesity patients. The bone areas and body circumferences were calculated by changing HU values between 120 and 2000 HU.

### 2.5. Regression Models for the Phantomless HU-to-BMD Conversion and Their Correlation Test

In this paper, patient physical data, HU values, and BMD values were considered for regression. Through univariate analyses of each factor, only the statistically significant factors were included in the subsequent multivariate analyses; consequently, the circumference, bone area, HU values, and BMD values were considered in the multivariate analyses. Through a stepwise regression method, the phantomless HU-to-BMD conversion equations were derived using multiple regression models. The independent variable was the BMD value; the dependent variables were the HU value, the circumference, and the bone area.

Pearson's correlation test was conducted in order to examine the correlation between the estimated BMD values and the reference values calculated using equation (1). All statistical analyses were performed using statistical software (R package 2.15.1). *P*-values of less than 0.05 were considered to be statistically significant.

### 2.6. Comparison of Voxelwise BMD

For the voxelwise comparison, the HU values of the spine and hip at the same axial level were reformatted to 512 × 512 array data. After both the estimated and reference BMD values were calculated, the deviation from the reference BMD values was measured in terms of the root-mean-square error (RMSE) as follows:

(2) $$\begin{array}{c} \text{RMSE}=\sqrt{\frac{{\sum_{i=1}^{n}\left({\left({\text{BMD}}_{\text{phantom}}\right)}_{i}-{\left({\text{BMD}}_{\text{phantomless}}\right)}_{i}\right)}^{2}}{n}}, \end{array}$$

where (BMD_phantom_)_*i*_ denotes the reference BMD value of the *i*th voxel calculated by equation (1) with the phantom; (BMD_phantomless_)_*i*_ is the BMD value estimated using the proposed phantomless conversion equations (equation (6)); and *n* indicates the total number of voxels. The conversion and statistical calculations were performed using the commercial software Interactive Data Language (IDL) (Exelis Vis Inc., Boulder, CO, USA).

### 2.7. Comparison of Finite Element Analysis Results

This study performed FE analysis to quantitatively investigate the effect of the estimated BMD on the structural behavior; the reference BMD was used as the ground truth. Segmentation of spine and hip images was first conducted using ITK-SNAP 3.6.0 [30]; hole filling and surface smoothing were implemented as postprocessing [31]. Then, each voxel in the segmented images was directly converted to a corresponding 8-node solid element. To assign the material properties required for the FE analysis, both the estimated and reference BMD values of each voxel were converted into the elastic moduli of each finite element by using the BMD-modulus relationship for the spine [32] and the femur [33] as follows:

(3) $$\begin{array}{c} \text{for}\;\;\mathrm{the}\;\;\mathrm{spine},\;\;E=5124\rho^{1.7}\;\;\left(\text{MPa}\right), \end{array}$$

(4) $$\begin{array}{c} \text{for}\;\;\mathrm{the}\;\;\mathrm{femur},\left\{\begin{array}{c} E=6850\rho^{1.49}\;\;\left(\mathrm{MPa}\right)\;\text{if}\;\;\rho \leq 1.64\;\;\left(\text{g}\cdot {\text{cm}}^{-3}\right),\\ E=4293\rho^{2.39}\;\;\left(\mathrm{MPa}\right)\;\text{if}\;\;\rho >1.64\;\;\left(\text{g}\cdot {\text{cm}}^{-3}\right), \end{array}\right. \end{array}$$

where *E* is Young's modulus of each finite element and *ρ* denotes the BMD of each voxel. For comparison, the linear volume fraction approach, which has been widely used due to easy implementation in the literature [34–37], was also used to assign the elastic modulus of each element as follows:

(5) $$\begin{array}{c} E=E_{0}\times \left(\text{BVF}\right)\;\;\left(\mathrm{MPa}\right), \end{array}$$

where (BVF) denotes the voxelwise volume fraction, which was linearly scaled to include the range of 0%–100% with the minimum and maximum values of voxel intensity for pure marrow and bone, respectively, in the CT images; *E*_0_ is the maximum elastic modulus obtained by the reference BMD (10.2 GPa for the spine and 8.4 GPa for the femur in this paper). Poisson's ratio was identically set to 0.3 for both the spine and femur. Pure compression and sideways falling conditions [33, 38] were selected as boundary conditions for the spine and femur, respectively. For the spine, a resultant force of 2,000 N was uniformly and vertically applied on the vertebral superior endplate. On the other hand, a vertical resultant force of 1000 N was applied in the distributed form towards the center of the femoral head. All FE analyses were conducted using the commercial software ANSYS 14.0.

## 3. Results

For the CT image of each patient, *α* and *β* were determined using the known mineral densities and corresponding HU values of the external phantom. After *α* and *β* in equation (1) were determined for the patient-specific calibration, the reference BMD values of the lumbar spine and hip for each patient were calculated using equation (1).

**Figure 2:**
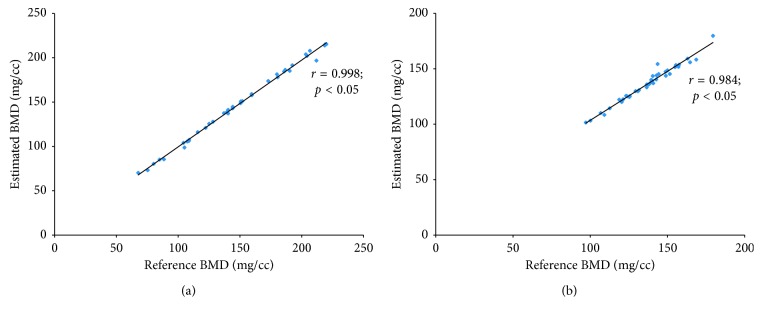
Pearson's correlation test of the estimated BMD values and those derived using a reference phantom: (a) Pearson's correlation coefficient of 0.998 for the L2 level, and (b) Pearson's correlation coefficient of 0.984 for the hip level (*p* < 0.05).

Through the univariate analyses, the HU value and circumference of the body were determined to be statistically significant (*p* < 0.05) for the lumbar spine; only the HU value was statistically significant (*p* < 0.05) for the hip. Note that the bone area was determined not to be significant (*p* > 0.05) for both the lumbar spine and hip. Therefore, the phantomless HU-to-BMD conversion equations for the lumbar spine and hip were derived from the multiple linear regression models as follows:

(6) $$\begin{array}{c} {\left(\text{BMD}\right)}_{\text{phantomless}}=0.848\times \left(\text{HU}\right)+0.148\times \left(\text{circumference}\right)-7.4\;\text{for}\;\;\mathrm{the}\;\;\mathrm{lumbar}\;\;\mathrm{spine,}\\ {\left(\text{BMD}\right)}_{\text{phantomless}}=0.784\times \left(\text{HU}\right)+16.6\;\text{for}\;\;\mathrm{the}\;\;\mathrm{hip,} \end{array}$$

where the units of BMD and circumference are mg/cc and cm, respectively. From Pearson's correlation test (Figure 2), it was verified that the BMD values estimated using equation (6) were significantly correlated with the reference BMD values from equation (1). Pearson's correlation coefficients were 0.998 and 0.984 for the lumbar spine and hip, respectively, and *p* < 0.05.

**Figure 3:**
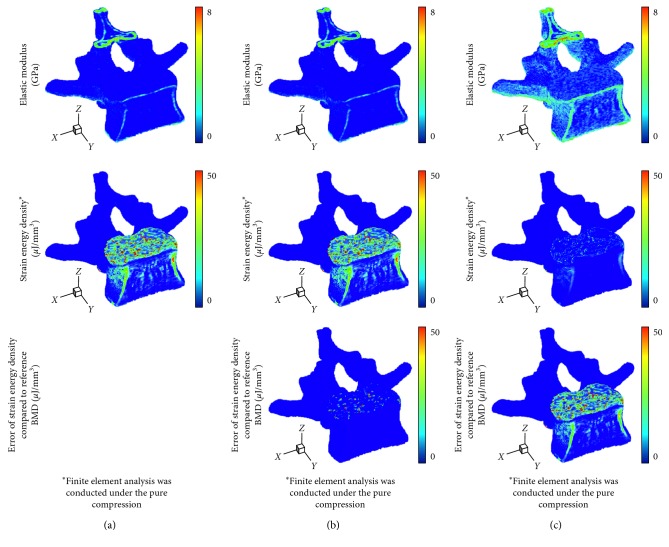
Distribution of elastic modulus, strain energy density, and error of strain energy density in the L2 vertebra: (a) case of using the reference BMD values with an external phantom, (b) case of using BMD values estimated by the proposed phantomeless HU-to-BMD conversion, and (c) case of using the simplified conversion of linear volume fraction approach.

**Figure 4:**
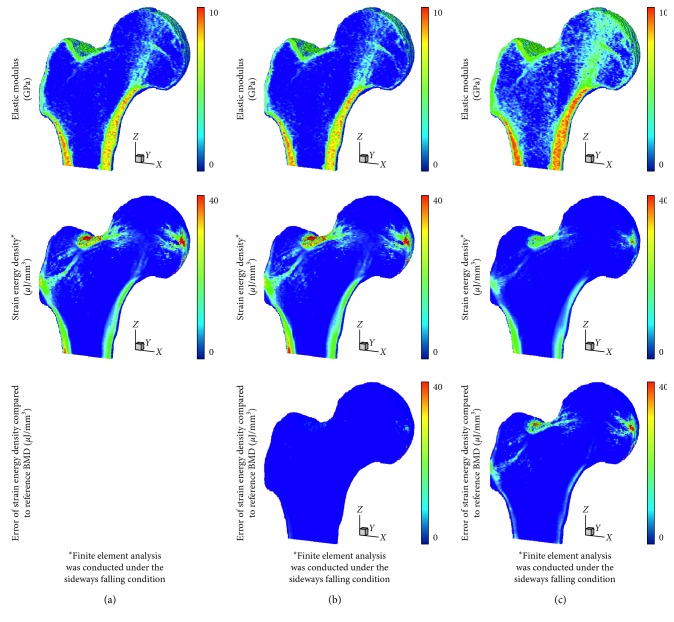
Distribution of elastic modulus, strain energy density, and error of strain energy density in the proximal femur: (a) case of using the reference BMD values with an external phantom, (b) case of using BMD values estimated by the proposed phantomeless HU-to-BMD conversion, and (c) case of using the simplified conversion of linear volume fraction approach.

In terms of the voxelwise BMD, the proposed phantomless HU-to-BMD conversion equations gave values of 4.26 ± 0.60 mg/cc and 8.35 ± 0.57 mg/cc for RMSE of the BMD deviation for the lumbar spine and hip, respectively. Figures 3 and 4 indicate that the distributions of the elastic modulus obtained using the estimated BMD values (793 ± 1174 MPa and 1330 ± 1747 MPa for the spine and hip, respectively) are almost identical to those obtained using the reference BMDs (818 ± 1231 MPa and 1473 ± 2023 MPa for the spine and hip, respectively).

Therefore, the FEA with the estimated BMD values delivered the average strain energy densities of 2.04 *μ*J/mm^3^ and 0.08 *μ*J/mm^3^ for the spine and hip, respectively, which are very close to those obtained with the reference BMDs (2.06 *μ*J/mm^3^ for the spine and 0.08 *μ*J/mm^3^ for the femur). In contrast, the elastic moduli estimated using the linear volume fraction approach (1756 ± 1492 MPa for the spine and 2146 ± 1967 MPa for the femur) were significantly higher than those obtained using the reference BMD values. Consequently, the average strain energy density for the linear volume fraction approach (0.51 *μ*J/mm^3^ for the spine and 0.03 *μ*J/mm^3^ for the femur) becomes much lower than that obtained using the reference BMD values. Note that total strain energy stored in the bone is inversely proportional to bone strength.

## 4. Discussion

BMD is a significant biomarker for bone strength [39] and therefore has an important function in the diagnostic criteria and therapeutic responses for osteoporosis. However, routine CT scans (i.e., CT scans without a reference phantom) do not directly provide the BMD values of a target bone; rather, they provide the HU values. With the recent trend of FEA-based quantitative assessment, this study was prompted by the necessity for reliable phantomless HU-to-BMD conversion, which includes the effects of patient-specific physical factors on the HU values. With 39 QCT images, the relationship between HU values and patient factors was investigated through multivariate analyses; linear regression models were obtained for phantomless HU-to-BMD conversion. A high positive correlation between the estimated and reference BMD values was found when using Pearson's correlation tests. From these results, it was concluded that BMD values of the lumbar spine can be estimated in terms of HU values and circumferences but that BMD values of the hip can be calculated using only the HU values. This difference of applicable regression models between the lumbar spine and hip may stem from the different amounts of soft tissues and bone areas at the abdominal level and at the hip level. However, for typical HU values of bone (700 to 1500 HU) and waist circumferences (65.941 to 97.356 cm in this study), the influence of waist circumference on the BMD is two orders of magnitude lower than that of HU. This implies that routine abdomen-pelvic or pelvic CT images (i.e., images obtained without using a reference phantom) have a high potential to be available for phantomless HU-to-BMD conversion through a process similar to the one proposed in this study. Recently, asynchronous calibration QCT has been introduced with excellent correlations, allowing for the quantification of BMD without the use of a calibration phantom [40, 41]. However, because the asynchronous phantomless QCT does not consider patient' factors which can affect HU values, it showed poor repeatability [42]. It would be more reliable to combine patient factors (e.g., size and body composition) with phantomless QCT.

**Figure 5:**
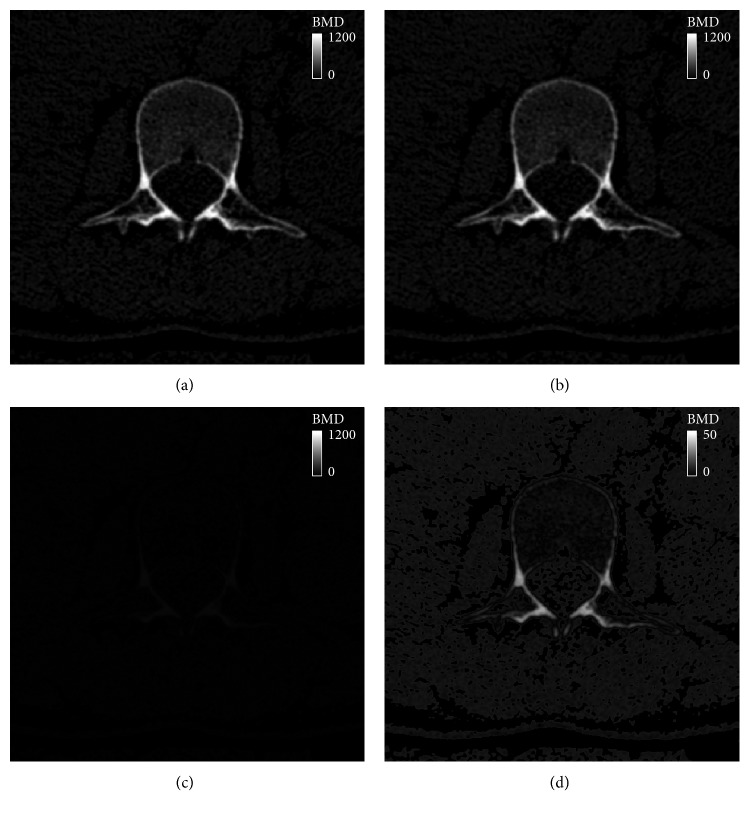
BMD contour plot at the axial level of the L2 vertebra: (a) the reference BMD using an external phantom, (b) BMD estimated using the proposed phantomless HU-to-BMD conversion, (c) a deviation between the reference and estimated BMD values, and (d) BMD deviation on a different scale.

**Figure 6:**
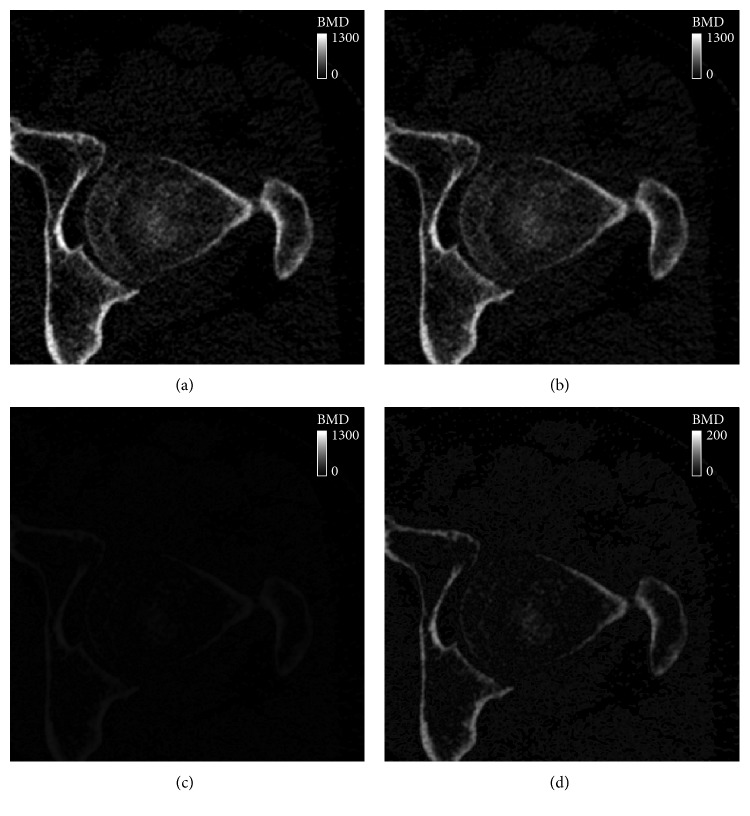
BMD contour plot at the axial level of the hip joint: (a) the reference BMD using an external phantom, (b) BMD estimated using the proposed phantomless HU-to-BMD conversion, (c) a deviation between the reference and estimated BMD values, and (d) BMD deviation on a different scale.

Figures 5 and 6 clearly show the similarity of the estimated and reference BMD values of each voxel. In terms of root-mean-square error (RMSE), the voxelwise BMD deviation (4.26 ± 0.60 mg/cc for the lumbar spine and 8.35 ± 0.57 mg/cc for the hip in this study) can be regarded negligible, compared with the maximum BMD (1000 mg/cc as shown in Figures 5(c) and 6(c)). Because an RMSE value can provide a representative value of the voxelwise BMD deviation for a region of interest, it can be concluded that the proposed phantomless HU-to-BMD conversion equations precisely estimate voxelwise BMD values for the lumbar spine and hip, thereby providing a reliable spatial BMD distribution in 3D. It is interesting to note that, as clearly depicted in Figures 5(d) and 6(d), the errors of the BMD values estimated using the proposed equations are linearly proportional, albeit negligibly, to their BMD values. Note that, for clearer visualization, the maximum BMD in the legend in Figures 5(d) and 6(d) was set to be different from that in Figures 5(c) and 6(c). This linear proportionality of BMD conversion errors stems from the statistically predetermined slope and *y*-intercept in equation (6), which are set in order to compensate for the patient-specific variation in attenuation.

As a more reliable tool for bone strength assessment [43], patient-specific FEA-based quantitative assessment requires voxelwise BMD data in order to assign the elastic modulus of each finite element through the BMD-modulus relationship. Patient-specific phantomless calibration of CT with an internal phantom has been also recently proposed to apply to preexisting clinical CT for quantitative bone densitometry and bone strength assessment for diagnostic and monitoring purposes [44]. However, when voxelwise BMD data were unknown due to no QCT examination, many studies have assumed that the elastic modulus of each finite element is linearly proportional to the volume fraction of voxel intensity with a range of 0 to 1 (thus, a range between the minimum and maximum values for pure marrow and bone, respectively) [34–37]. This simplified approach can cause non-negligible errors in the FEA results, as clearly shown in Figures 3 and 4. Considering that the BMD-modulus relationship for the spine and femur, validated in the literature [45, 46], is typically nonlinear, a simplified linear proportionality (equation (5) in this paper) causes the intermediate elastic moduli to become higher than those derived by the BMD-modulus relationship (equations (3) and (4) in this paper) when the maximum elastic moduli are set to be the same. Consequently, apparent bone stiffness becomes higher (i.e., lower total strain energies stored in Figures 3(c) and 4(c)) than it actually should be. In contrast, the voxelwise BMD values that were estimated using the proposed phantomless conversion led to reliable FEA results, which were nearly identical to those obtained with the aid of QCT examination. These results demonstrate the potential of the proposed phantomless HU-to-BMD conversion to conduct reliable FEA-based quantitative assessment using routine CT images without the aid of QCT examination. Accurate voxelwise BMD estimation is also essential for the bone microstructure reconstruction approach recently proposed [47, 48].

The limitations of this study should be addressed. The proposed phantomless HU-to-BMD equation is based on the QCT protocol: 120 kVp, 150 effective mAs, 3 mm slice thickness, and B40s medium kernel. For better accessibility, the proposed formula should be extended to include routine CT protocols. However, considering the radiation dose modulation techniques, the simple equations of kVp or mAs might not be adequate. Furthermore, the influence of the increased density of the contrast media was not evaluated. Because the CT density depends on the phase after the contrast injection, phantomless BMD estimation should be undertaken carefully when using contrast-enhanced CT images. Finally, although it has been reported that distinct CT scanners with the same acquisition protocol can have different scan data [49], this study used only a single kind of CT scanner due to a lack of availability. Therefore, the proposed method should be revisited more in depth for other kinds of CT scanners.

With follow-up research, the proposed models for phantomless HU-to-BMD conversion could be extended to utilize routine CT images. Because routine CT scans are performed in daily practice, the proposed model would enable patients to avoid additional radiation exposure for BMD measurement. Furthermore, FEA-based quantitative assessment would be available for osteoporosis study of large populations, which can provide more reliable diagnostic data in translational medicine.

## Data Availability

The QCT scan data used to support the findings of this study have not been made available because internal data research board does not permit data sharing without specific agreement.
